# Bone morphogenetic protein 4-associated mitochondrial dysfunction in diabetic nephropathy involves Parkin downregulation

**DOI:** 10.3389/fcdhc.2026.1860424

**Published:** 2026-08-11

**Authors:** Masanori Tamaki, Tatsuya Tominaga, Kazuhiro Hasegawa, Takahiro Kida, Shinji Miyakami, Ikuko Shimizu, Sumiyo Yamaguchi, Masanori Minato, Kojiro Nagai, Shu Wakino

**Affiliations:** 1 Department of Nephrology, Tokushima University Graduate School of Biomedical Sciences, Tokushima, Japan; 2 Department of Bioanalytical Technology, Tokushima University Graduate School of Biomedical Sciences, Tokushima, Japan; 3 Department of Nephrology, Shizuoka General Hospital, Shizuoka, Japan

**Keywords:** BMP4, diabetic nephropathy, mesangial cell, mitochondrial dysfunction, parkin

## Abstract

**Background:**

Diabetic nephropathy (DN) is characterized by mesangial expansion and mitochondrial dysfunction. Although bone morphogenetic protein 4 (BMP4) is a pro-fibrotic factor in DN, its role in mesangial mitochondrial homeostasis remains unclear. This study investigated whether a decrease in Parkin is involved in mitochondria-related abnormalities following BMP4 stimulation.

**Methods:**

Renal tissues from streptozotocin (STZ)-induced diabetic mice were subjected to biochemical, histological, and molecular analyses. *In vitro*, cultured mouse mesangial cells were exposed to recombinant BMP4 in the presence or absence of the activin receptor-like kinase (ALK) inhibitor LDN-193189. A mesangial cell line overexpressing Parkin was used to assess the role of Parkin under BMP4 stimulation. Mitochondrial function and the expression of Parkin and related genes were analyzed.

**Results:**

In STZ-induced diabetic mice, mesangial expansion was accompanied by mitochondrial abnormalities, increased BMP4 expression, diminished mtDNA copy number, and decreased expression of Parkin and related genes. In cultured mesangial cells, BMP4 stimulation was accompanied by a decrease in Parkin expression and reduced mitochondria-related indices, including decreased expression of mitochondrial function-related genes, reduced ATP production, and loss of membrane potential. The BMP4-induced changes in gene expression and mitochondrial functional indices were ameliorated by ALK inhibition. Furthermore, Parkin overexpression ameliorated BMP4-induced decreases in gene expression and mitochondrial functional indices.

**Conclusion:**

These findings suggest that decreased Parkin expression associated with BMP4 stimulation may be one of the molecular alterations contributing to mitochondria-related abnormalities in mesangial cells. Disrupted mitochondrial homeostasis accompanied by decreased Parkin expression may represent a candidate therapeutic pathway in DN.

## 1 Introduction

Diabetic nephropathy (DN) is a leading cause of end-stage renal disease; as such, further elucidation of its pathogenesis and subsequent development of novel therapeutic strategies are urgent issues (1–3). DN progression is characterized by mesangial matrix expansion, accompanied by the excessive accumulation of extracellular matrix in the glomerulus, which leads to glomerulosclerosis and nephron loss (1, 2). In mesangial cells, which maintain glomerular structure and filtration function, energy metabolism via the mitochondria is considered important for maintaining normal cellular functions. Recent reports have indicated that the disruption of mitochondrial homeostasis and functional decline occur in mesangial cells under diabetic conditions (1, 3). Abnormalities in mitochondrial homeostasis have been suggested to be involved in mesangial cell injury via abnormal energy metabolism, such as decreases in ATP production, and may promote DN progression (1, 3).

Bone morphogenetic protein 4 (BMP4), a pro-fibrotic factor belonging to the TGF-β superfamily, is upregulated in renal tissues under diabetic conditions (4–6). Our previous studies demonstrated that BMP4 acts on mesangial cells via Smad1 signaling and other pathways to promote the production of matrix proteins, such as type IV collagen, thereby driving mesangial matrix expansion (4–6). Furthermore, recent studies on other cell types, such as the whitening of brown adipocytes (7) and vascular endothelial cell injury in diabetic retinopathy (8), have increasingly reported that enhanced BMP4 signaling is associated with mitochondrial morphological abnormalities and functional decline. However, whether BMP4 is involved in mitochondrial functional abnormalities in mesangial cells in DN, beyond its known fibrosis-related effects, remains unclear.

Mitochondrial quality and quantity are maintained through a balance between mitophagy and biogenesis (9). Parkin is known to be involved in the removal/mitophagy of damaged mitochondria and is reportedly associated with mitochondrial biogenesis-related pathways (10). As such, Parkin is considered to play an important role in mitochondrial quality control (11). Although decreased Parkin expression has been reported in clinical and experimental studies of DN (12–14), whether this decrease occurs downstream of BMP4 signaling and whether the maintenance of Parkin expression can improve BMP4-induced mitochondria-related abnormalities remain unclear.

In the context of previously unexplained mitochondrial dysfunction of mesangial cells in DN, the present study aimed to investigate whether the decrease in Parkin expression following BMP4 stimulation is involved in mitochondria-related abnormalities in mesangial cells. To address this issue, we used an *in vivo* streptozotocin (STZ)-induced diabetes mouse model and an *in vitro* model of cultured mesangial cells exposed to BMP4. Furthermore, we examined the role of Parkin under BMP4 stimulation and its effects on mitochondria-related indices using an activin receptor-like kinase (ALK) inhibitor and Parkin overexpression. Because BMP4 is upregulated within the diabetic milieu (4–6), recombinant BMP4 was used to isolate its specific contribution as a downstream mediator, complementary to the *in vivo* diabetic model.

## 2 Materials and methods

### 2.1 Experimental animals and diabetes model

Male Crl: CD1(ICR) mice were purchased from Charles River Laboratories (Tokyo, Japan), and maintained under specific pathogen-free conditions at the Tokushima University Animal Facility. A low-dose, multiple-STZ injection model was applied to 8-week-old male ICR mice according to a previously described protocol (15). In brief, STZ (50 mg/kg BW, #195-15154, Wako Pure Chemical Industries, Osaka, Japan) dissolved in freshly prepared 0.1 M citrate buffer (pH 4.5) was intraperitoneally administered for 5 consecutive days (16). Fasting blood glucose levels were measured 4 weeks following the first STZ injection (at 12 weeks of age), and mice exhibiting sustained hyperglycemia (≥ 400 mg/dL) were defined as the diabetic group and used for all subsequent analyses. All animals were euthanized 6 months following the initial STZ or vehicle injection (at approximately 34 weeks of age). Mice were deeply anesthetized with inhaled isoflurane (5%) and euthanized by exsanguination via the inferior vena cava. Renal tissues were immediately harvested for biochemical, histological, and molecular biological evaluations.

### 2.2 Biochemical and histological analysis

To measure albuminuria, 24-hour urine samples were collected in metabolic cages. Urinary albumin concentrations were measured using the Albuwell Kit (#1011, Exocell, a brand of Ethos Biosciences, Logan Township, NJ, USA), and the daily albumin excretion rate (µg/day) was calculated based on the 24-hour urine volume.

For histological evaluation, renal tissues were fixed in Carnoy’s solution and embedded in paraffin. Sections (2 µm thick) were prepared and subjected to periodic acid-methenamine silver (PAM) staining. Quantification of mesangial matrix expansion was conducted by an examiner blinded to group assignments. At least 20 cortical glomeruli were randomly selected from each mouse and imaged under a light microscope. The total glomerular area and PAM-positive area were measured using ImageJ software (National Institutes of Health, Bethesda, MD, USA), and the percentage of PAM-positive area was calculated (6).

### 2.3 Cell culture and treatments

Cultured mouse mesangial cells were established from the glomeruli of Crl: CD1(ICR) mice as previously described (6, 17, 18). The cells were maintained and cultured in B medium (a 3:1 mixture of MEM and F12) supplemented with 20% fetal calf serum (FCS), 1 mM glutamine, 100 U/mL penicillin, and 100 µg/mL streptomycin. For the experiments, cells were starved in B medium containing 0.2% FCS and then treated with recombinant BMP4 (15 ng/mL, #HZ-1045, Proteintech, Rosemont, IL, USA) for 24 h. In receptor inhibition experiments, an ALK2/3- selective inhibitor, LDN-193189 (100 nM, #HY-12071A, MedChemExpress, Monmouth Junction, NJ, USA), was added to the cells with BMP4 (19). For high-glucose experiments, cells were cultured in normal-glucose B medium or high-glucose (30 mM D-glucose) medium for 48 h, with mannitol added to the normal-glucose medium as an osmotic control; mitochondrial membrane potential and ATP production were then evaluated as described below.

### 2.4 siRNA transfection and stable overexpression

For transient knockdown of Parkin, a specific siRNA targeting *Park2* (10 nM, Silencer Select siRNA #4390771, Thermo Fisher Scientific, Waltham, MA, USA) or control siRNA (10 nM, #4390843, Thermo Fisher Scientific) was transfected into mesangial cells at 60–80% confluence using Lipofectamine RNAiMAX (#13778030, Thermo Fisher Scientific). After transfection, cells were cultured in B medium containing 10% FCS for 48 h, followed by culture in B medium containing 0.2% FCS for 24 h before use in various assays.

To establish a cell line constitutively overexpressing (OE) Parkin, a control vector (CoralHue™ Mitochondria-targeted mKeima-Red, #AM-V0251M, MBL Life Science, Tokyo, Japan) or a *Park2* expression vector (pMitophagy Keima-Red mPark2, #AM-V0259M, MBL Life Science) was transfected into mesangial cells using Lipofectamine 3000 (#L3000008, Thermo Fisher Scientific). Transfected cells were selected using the antibiotic G-418 Sulfate (#V8091; Promega, Madison, WI, USA). Following G418 selection, two independent clones were established for each of the *Park2* expression vector and the control vector. Overexpression of Parkin protein was confirmed in both Parkin-OE clones by Western blotting. For the subsequent functional and gene-expression experiments, one clone showing stable and sufficient overexpression was used, together with the corresponding empty-vector control clone.

### 2.5 Evaluation of mitochondrial function

Mitochondrial function was evaluated according to the manufacturer’s instructions. Mitochondrial membrane potential (ΔΨm) was assessed using the JC-1 MitoMP Detection Kit (#MT09, Dojindo, Kumamoto, Japan). For representative imaging, JC-1 fluorescence was acquired on a Keyence BZ-9000 (PlanApo 20×/NA 0.75) under blinded conditions using identical exposure and detector settings across all groups, and the red (J-aggregate) and green (monomer) channels were merged in ImageJ 1.54 with identical linear display settings. For quantitative assessment, red (J-aggregate) and green (monomer) fluorescence intensities were measured using a multi-mode microplate reader (GENios; Tecan, Männedorf, Switzerland), and ΔΨm was expressed as the red-to-green fluorescence intensity ratio relative to the control group. Intracellular ATP production was quantified by chemiluminescence assay using an ATP detection assay kit (#700410; Cayman Chemical, Ann Arbor, MI, USA) and normalized to the cell number calculated using a Cell Count Normalization Kit (#C544; Dojindo).

To measure cytochrome c oxidase (COX) activity, mitochondrial fractions were isolated using a Mitochondrial Isolation Kit for Tissue and Cultured Cells (#KC010100; BioChain, Newark, CA, USA). Subsequently, cytochrome c oxidase activity was evaluated spectrophotometrically using a mitochondrial activity assay kit (#KC310100, BioChain).

Cellular oxygen consumption was measured using the MitoXpress Xtra Oxygen Consumption Assay (Agilent Technologies, Santa Clara, CA, USA), and the intracellular antioxidant capacity was evaluated in cell extracts using the Antioxidant Assay Kit (#709001, Cayman Chemical) based on the ABTS method. Absorbance, fluorescence, and chemiluminescence were measured using a multi-mode microplate reader (GENios; Tecan, Männedorf, Switzerland).

### 2.6 Gene and protein expression analysis

RNA extraction and real-time PCR were performed as described below. Total RNA was extracted using the RNeasy Mini Kit (#74104; Qiagen, Hilden, Germany). cDNA was synthesized using the PrimeScript RT reagent Kit (#RR037A, Takara Bio, Shiga, Japan), and quantitative PCR was conducted using a real-time PCR system (MiniOpticon, Bio-Rad, Hercules, CA, USA) with GoTaq^®^ qPCR Master Mix (#A6001, Promega) and specific primers. Relative mRNA expression levels were calculated by the ΔΔCt method using *Actb* as an internal control. The primer sequences are listed in **Supplementary Table 1** (20–24).

To evaluate the mitochondrial DNA (mtDNA) copy number, total DNA was extracted from cells and tissues using the DNeasy Blood & Tissue Kit (#69504, Qiagen), and quantitative PCR was conducted using a mitochondrial gene (*Nd1*) and a nuclear gene (*Hk2*) as internal controls (24). The mtDNA copy number was calculated relative to the nuclear genome.

For Western blot analysis, renal tissues were homogenized in RIPA buffer supplemented with a protease inhibitor cocktail, and total protein was extracted from cells using a Mammalian Cell Extraction Kit (#K269-500, BioVision, Milpitas, CA, USA). Protein concentrations were measured and equalized using a Qubit 1.0 Fluorometer (Thermo Fisher Scientific). Equal quantities of protein were separated by SDS-PAGE and transferred to PVDF membranes using a Trans-Blot Turbo Transfer System and dedicated packs (#1704156; Bio-Rad). After blocking with the ECL Prime Blocking Agent (Cytiva, Marlborough, MA, USA), membranes were incubated with primary antibodies. The primary antibodies used were: anti-Parkin mouse mAb (Prk8, #4211) and anti-TOM20 rabbit mAb (D8T4N, #42406) (both from Cell Signaling Technology, Danvers, MA, USA), with anti-β-actin (#A5316, Sigma-Aldrich, St. Louis, MO, USA) and anti-α-tubulin (#T6199, Sigma-Aldrich) used as internal controls. After washing, membranes were incubated with horseradish peroxidase-conjugated secondary antibodies (anti-rabbit IgG, #17502, Immuno-Biological Laboratories, Gunma, Japan; and anti-mouse IgG1, ab99617, Abcam, Cambridge, UK). Chemiluminescent signals were finally visualized using Immobilon ECL Ultra Western HRP Substrate (no. WBULS0100; Merck Millipore, Burlington, MA, USA) and acquired using an image analysis system (LAS-3000; Fujifilm, Tokyo, Japan). Band intensities were quantified using ImageJ software.

### 2.7 Statistical analysis

All data are presented as the mean ± standard error of the mean (SEM). Comparisons between the two groups were conducted using the unpaired Student’s t-test. One-way analysis of variance (ANOVA) was used, followed by the Tukey-Kramer method for comparisons among three or more groups. All statistical analyses were performed using a Bell Curve for Excel software (version 4.05; Social Survey Research Information Co., Ltd., Tokyo, Japan). Statistical significance was set at a *P*-value < 0.05.

### 2.8 Ethical approval

All animal experimental procedures were conducted in accordance with the Guidelines for Animal Experimentation of Tokushima University, applicable national laws and regulations, and the National Institutes of Health Guide for the Care and Use of Laboratory Animals. All animal experiments were approved by the Institutional Animal Care and Use Committee of Tokushima University (Approval Nos. T2020–119 and T2024-1).

## 3 Results

### 3.1 Renal injury and alterations in mitochondria-related indices in a diabetic nephropathy model (STZ Mice)

**Figure 1:**
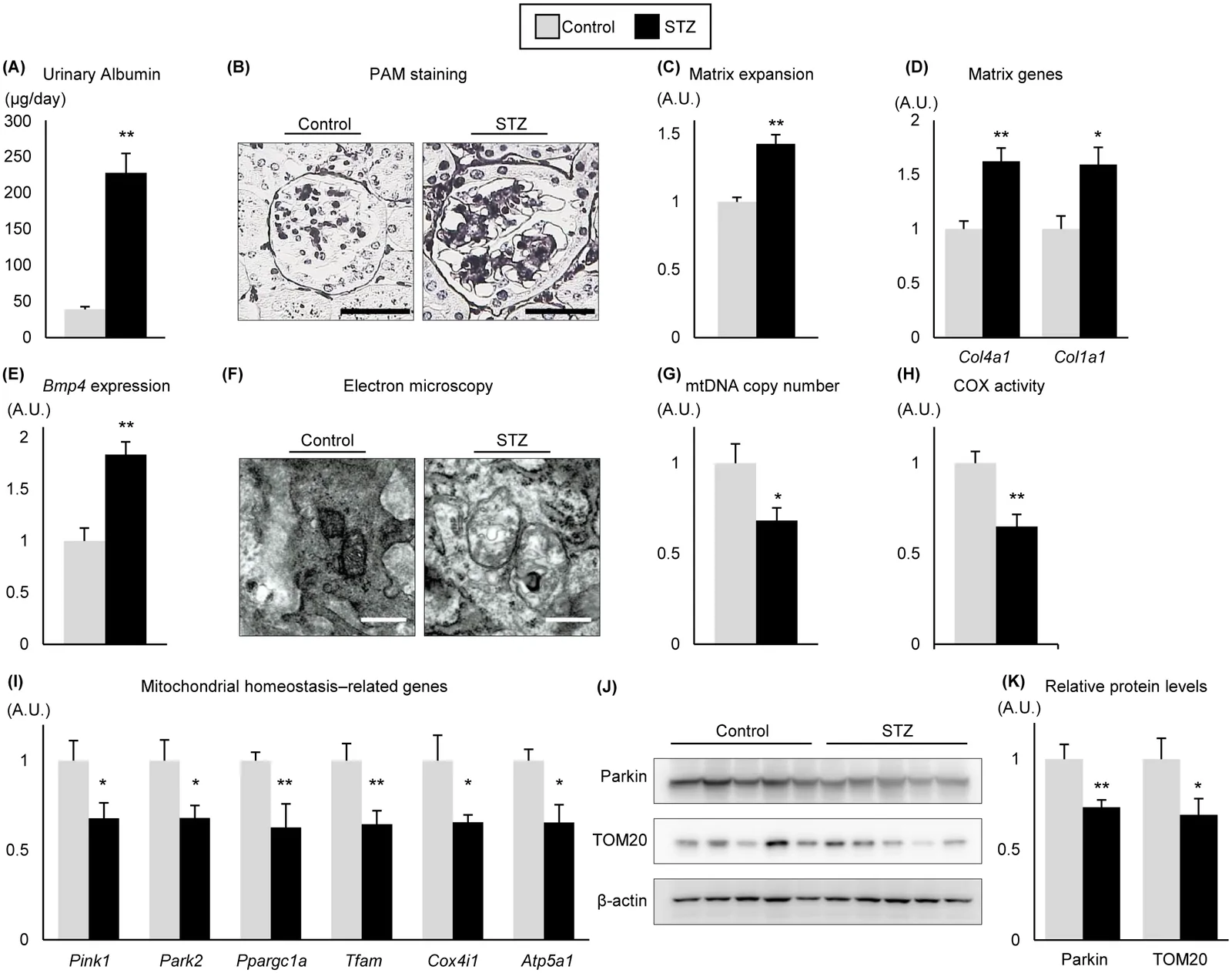
Diabetic nephropathy model (STZ-treated) mice show mitochondrial dysfunction and decreased Parkin expression. **(A)** Urinary albumin excretion in control and STZ-treated groups (6 months post-administration). **(B)** Representative PAM staining images of renal tissues. **(C)** Quantitative evaluation of mesangial matrix expansion. **(D)** mRNA expression levels of the extracellular matrix-related genes *Col4a1*, *Col1a1*. **(E)** mRNA expression levels of *Bmp4*. **(F)** Representative transmission electron microscopy images of renal tissues, showing mitochondrial swelling and disrupted cristae in the STZ group. **(G)** mtDNA copy number (*Nd1*) in the renal tissues. **(H)** COX activity. **(I)** mRNA expression profiles of the mitochondrial homeostasis-related genes *Pink1*, *Park2*, *Ppargc1a*, *Tfam*, *Cox4i1*, and *Atp5a1*. **(J)** Representative western blot images of Parkin and TOM20 (with β-actin used as an internal control) and **(K)** their quantitative results. Data are presented as the mean ± SEM (n = 10 in each group). *P < 0.05, **P < 0.01 vs. Control.

We used an STZ-induced diabetic mouse model to evaluate the relationship between DN pathology and mitochondrial-related indices *in vivo*. The STZ-treated group showed an increase in urinary albumin excretion compared to the control group (**Figure 1A**). Histological analysis using PAM staining revealed mesangial matrix expansion in the STZ group (**Figures 1B, C**). Further, the mRNA expression levels of the extracellular matrix (ECM)-related genes *Col4a1* and *Col1a1* were upregulated in the STZ group (**Figure 1D**). Consistent with previous reports (4–6), we additionally confirmed a significant increase in *Bmp4* mRNA expression in the renal tissues of the STZ group (**Figure 1E**).

Next, we evaluated the morphology and function of mitochondria in renal tissues. Electron microscopic observation revealed morphological changes in the mitochondria of the mesangial cells of the STZ group, such as mitochondrial swelling and disrupted cristae structures (**Figure 1F**). Furthermore, both the mtDNA copy number (**Figure 1G**) and COX activity (**Figure 1H**) were decreased in the STZ group.

To investigate the molecular indices associated with these alterations, we analyzed the expression of mitochondrial homeostasis-related genes. The results revealed decreased mRNA expression of *Pink1* and *Park2* in the STZ group (**Figure 1I**). Moreover, the expression of *Ppargc1a* and its related genes (*Tfam*, *Cox4i1*, and *Atp5a1*) was downregulated (**Figure 1I**). The protein expression of Parkin and the outer mitochondrial membrane protein TOM20 decreased in the STZ group (**Figures 1J, K**). These results indicated that, along with renal injury, STZ mouse kidneys exhibited a decrease in Parkin-related molecules and mitochondria-related indices.

### 3.2 BMP4 exposure is accompanied by decreased mitochondria-related indices in cultured mesangial cells

**Figure 2:**
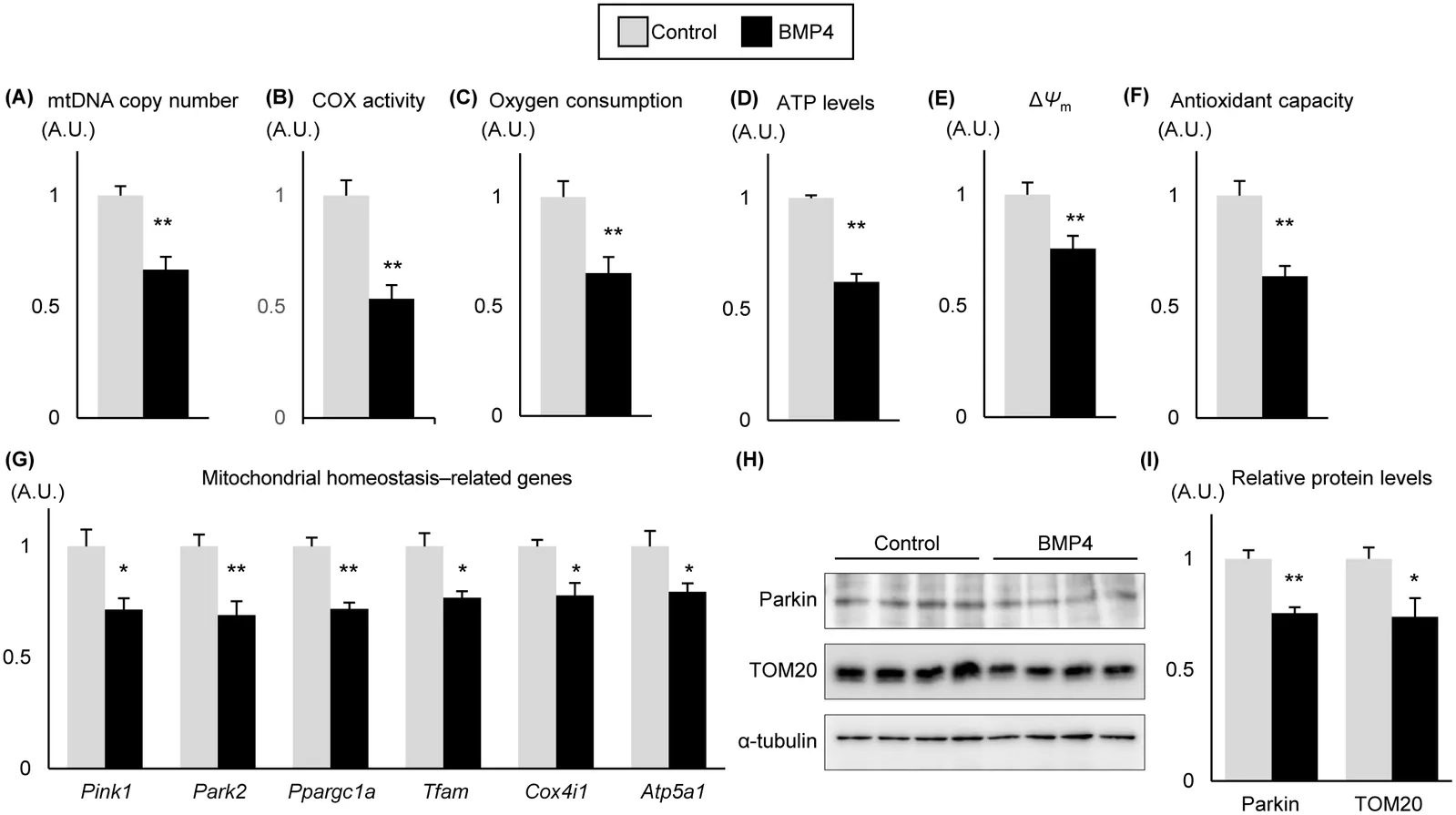
Cultured mesangial cells show BMP4-induced mitochondrial abnormalities and alterations in gene expression. Cultured mesangial cells were treated with recombinant BMP4 (15 ng/mL) for 24 h and subsequently evaluated to assess: **(A)** mtDNA copy numbers, **(B)** COX activity, **(C)** Oxygen consumption, **(D)** ATP production, **(E)** Mitochondrial membrane potential (ΔΨm), **(F)** Antioxidant capacity (ABTS assay), **(G)** mRNA expression levels of mitochondrial homeostasis-related genes, **(H)** Representative western blot images of Parkin and TOM20 (using α-tubulin as an internal control), and **(I)** their quantitative results. Data are presented as the mean ± SEM of 3 independent experiments. *P < 0.05, **P < 0.01 vs. Control.

Next, to investigate the relationship between BMP4, whose expression is upregulated in DN, and mitochondria-related abnormalities, we conducted an *in vitro* analysis using cultured mesangial cells. We first confirmed that high glucose itself impairs mesangial mitochondrial function: compared with the osmotic (mannitol) control, high-glucose stimulation reduced ATP production and mitochondrial membrane potential (**Supplementary Figure 1**), consistent with previous reports in human mesangial cells (25). We then examined BMP4 as a specific upstream mediator. BMP4 stimulation decreased the mtDNA copy number (**Figure 2A**) and COX activity (**Figure 2B**). Additionally, oxygen consumption (**Figure 2C**), intracellular ATP levels (**Figure 2D**), mitochondrial membrane potential (ΔΨm) (**Figure 2E**), and antioxidant capacity (**Figure 2F**) were also reduced by BMP4 stimulation. Gene expression analysis revealed that BMP4 stimulation decreased the mRNA expression of *Pink1* and *Park2*. The expression of *Ppargc1a* and its related genes (*Tfam*, *Cox4i1*, and *Atp5a1*) was also downregulated (**Figure 2G**). In addition, BMP4 stimulation decreased the protein expression of Parkin and TOM20 (**Figures 2H, I**). These results demonstrate that BMP4 stimulation in cultured mesangial cells was accompanied by decreases in mitochondria-related functional indices and expression of Parkin-related molecules.

### 3.3 Parkin knockdown induces mitochondria-related alterations in cultured mesangial cells

**Figure 3:**
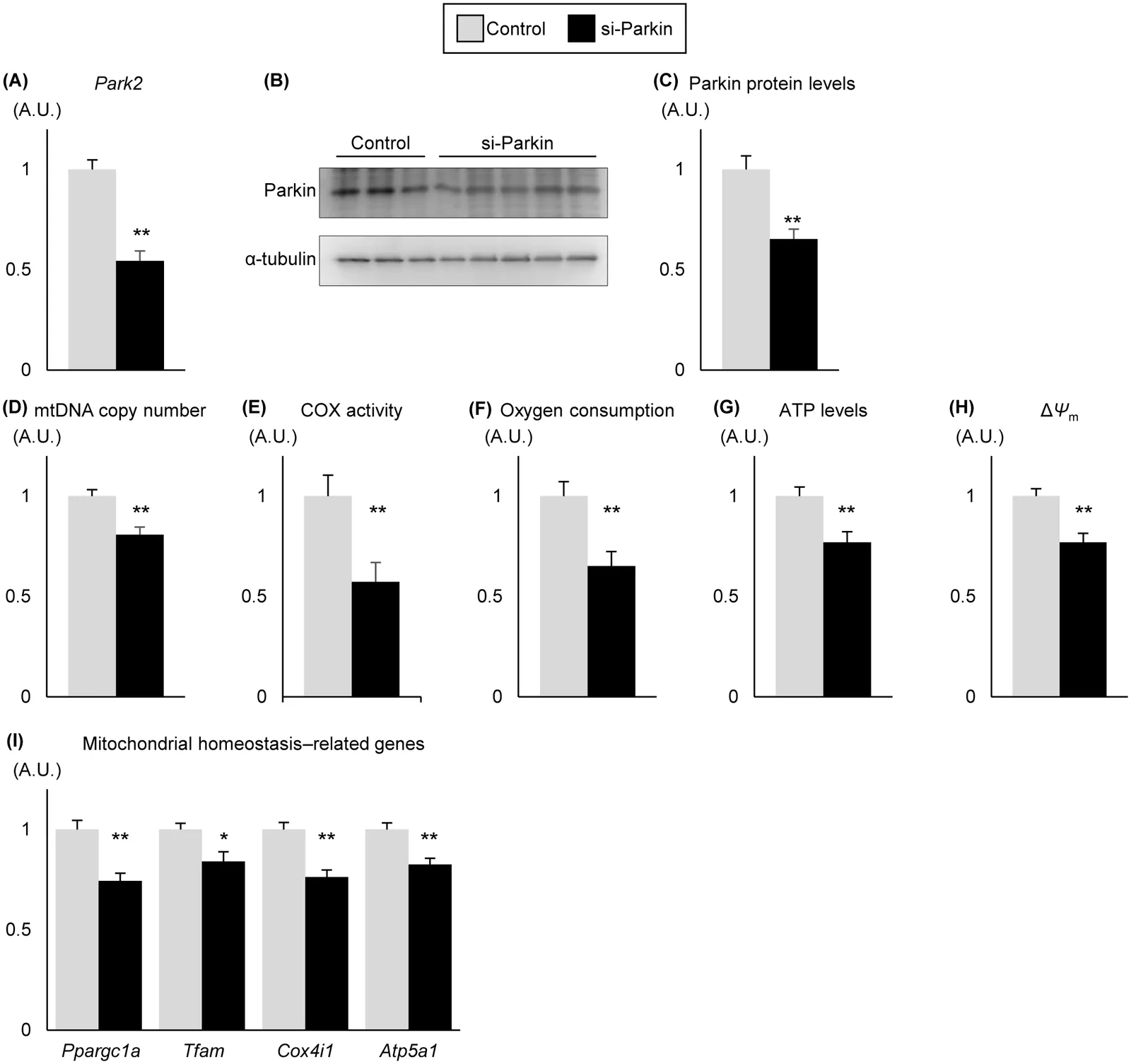
Parkin knockdown impairs mitochondrial function and the expression of PGC-1α-related genes. Cultured mesangial cells were transfected with *Park2*-specific (10 nM) or control siRNA and evaluated after 48 h of normal culture, followed by 24 h of starvation (72 h in total). **(A)** *Park2* mRNA expression. **(B)** Representative western blot images of Parkin (using α-tubulin as an internal control) and **(C)** quantification of protein levels. **(D)** mtDNA copy numbers. **(E)** COX activity. **(F)** Oxygen consumption. **(G)** ATP production. **(H)** ΔΨm. **(I)** mRNA expression levels of mitochondrial biogenesis-related genes (*Ppargc1a*, *Tfam*, *Cox4i1*, *Atp5a1*). Data are presented as the mean ± SEM of 3 independent experiments. *P < 0.05, **P < 0.01 vs. Control.

To further investigate the relationship between the Parkin reduction observed in both the *in vivo* and *in vitro* models and mitochondria-related abnormalities, we induced Parkin knockdown (KD) in cultured mesangial cells using siRNA. The introduction of siRNA resulted in a clear reduction in *Park2* mRNA expression and was accompanied by a reduction in Parkin protein signal (**Figures 3A–C**). Compared to control cells, cells with Parkin KD exhibited decreases in mtDNA copy number (**Figure 3D**), COX activity (**Figure 3E**), oxygen consumption (**Figure 3F**), intracellular ATP levels (**Figure 3G**), and ΔΨm (**Figure 3H**). Furthermore, gene expression analysis revealed that Parkin KD decreased the mRNA expression of *Ppargc1a* and mitochondria-related genes (*Tfam*, *Cox4i1*, and *Atp5a1*) (**Figure 3I**). These changes were similar to those observed following BMP4 stimulation (**Figure 2**). These results indicate that the reduction in Parkin is associated with a decrease in mitochondria-related gene expression and functional indices in cultured mesangial cells.

### 3.4 Mitochondria-related alterations induced by BMP4 stimulation are ameliorated by ALK inhibition and parkin overexpression

**Figure 4:**
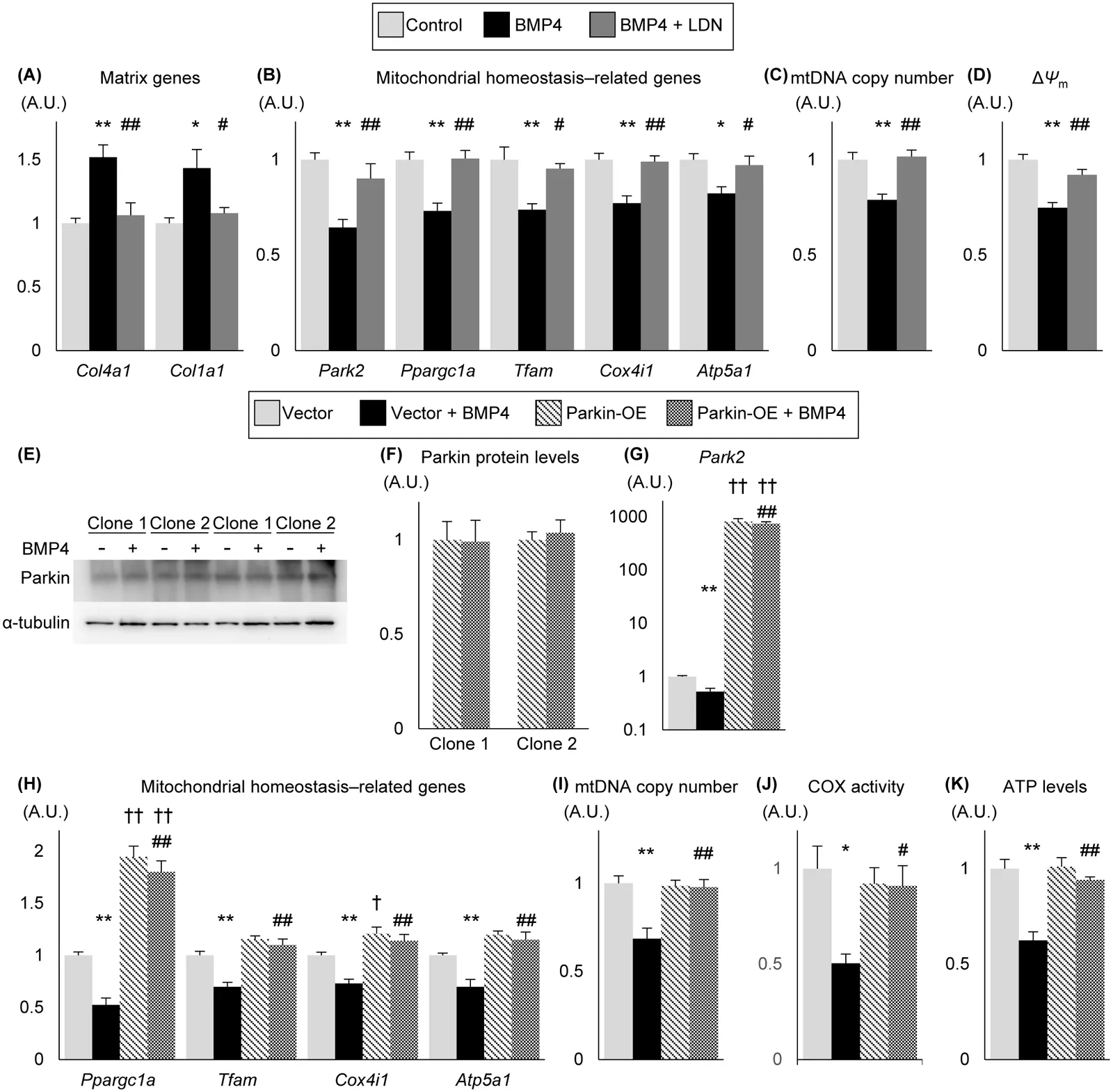
ALK inhibition and Parkin overexpression rescue BMP4-induced mitochondrial impairment. **(A–D)** Effects of an ALK inhibitor: Cultured mesangial cells were co-treated with the ALK2/3 inhibitor LDN-193189 (100 nM) and BMP4 (15 ng/mL) for 24 h. **(A)** mRNA expression of matrix genes (*Col4a1* and *Col1a1*). **(B)** mitochondria-related genes. **(C)** The mtDNA copy number. **(D)** ΔΨm. Data are presented as the mean ± SEM of 3 independent experiments. *P < 0.05, **P < 0.01 vs. Control; #P < 0.05, ##P < 0.01 vs. BMP4 alone. **(E–K)** Effects of Parkin overexpression (OE): Mesangial cells constitutively overexpressing Parkin (Parkin OE) or cells transfected with a control vector (Vector) were treated with BMP4 (15 ng/mL) for 24 h. **(E)** Representative western blot images of Parkin (using α-tubulin as an internal control) in Parkin-OE cells (clone 1 and clone 2, each with two independently cultured samples) treated with or without BMP4, and **(F)** quantification of Parkin protein normalized to α-tubulin and expressed relative to the untreated sample within each clone. **(G)** *Park2* mRNA expression (logarithmic axis). **(H)** mRNA expression levels of mitochondrial biogenesis-related genes. **(I)** mtDNA copy numbers. **(J)** COX activity. **(K)** ATP production. Data are presented as the mean ± SEM of 3 independent experiments. *P < 0.05, **P < 0.01 vs. Vector control group; ^#^P < 0.05, ^##^P < 0.01 vs. Vector + BMP4 group; ^†^P < 0.05, ^††^P < 0.01 vs. Vector control (in Parkin-OE groups).

Next, we investigated the involvement of ALK signaling in Parkin reduction and mitochondria-related alterations induced by BMP4 stimulation, as well as the effects of Parkin overexpression. First, cultured mesangial cells were treated with BMP4 and the ALK inhibitor LDN-193189. Upregulation of the mRNA expressions of extracellular matrix-related genes (*Col4a1* and *Col1a1*) induced by BMP4 was suppressed by treatment with LDN-193189 (**Figure 4A**). Furthermore, the downregulation of *Park2*, *Ppargc1a*, and mitochondria-related genes (*Tfam*, *Cox4i1*, *Atp5a1*) (**Figure 4B**), as well as the decreases in mtDNA copy number (**Figure 4C**) and ΔΨm (**Figure 4D**) caused by BMP4 stimulation, were ameliorated by LDN-193189 treatment. Representative JC-1 images of these groups showed a predominance of green monomers after BMP4 and recovery of red J-aggregate puncta with LDN-193189, consistent with the ratiometric ΔΨm quantification (**Supplementary Figure 2**). These results indicate that ALK signaling may be involved in the changes in Parkin-related molecules and mitochondria-related indices induced by BMP4 stimulation.

Finally, to examine the role of Parkin, we evaluated the effect of BMP4 stimulation on mesangial cells constitutively overexpressing Parkin. Marked Parkin protein overexpression in the established clones was confirmed by Western blotting. Because of this strong overexpression, a quantitative comparison of Parkin protein between vector and Parkin-OE cells under identical exposure conditions was not appropriate; however, comparison within each Parkin-OE clone under non-saturating conditions showed that Parkin protein in Parkin-OE cells was not reduced by BMP4 (**Figures 4E, F**), whereas BMP4 reduced endogenous Parkin in mesangial cells (**Figures 2H, I**). In vector cells, BMP4 reduced *Park2* mRNA, whereas *Park2* remained markedly elevated in Parkin-OE cells (**Figure 4G**). In empty vector-transfected cells (vector), BMP4 stimulation reduced the mRNA expression of *Ppargc1a* and its related genes, while this downregulation was reversed in Parkin-OE cells (**Figure 4H**). Additionally, the decrease in mtDNA copy number (**Figure 4I**), COX activity (**Figure 4J**), and intracellular ATP levels (**Figure 4K**) induced by BMP4 in Vector cells were restored in Parkin-OE cells. These results demonstrate that Parkin overexpression ameliorated the decrease in mitochondria-related gene expression and functional indices induced by BMP4 stimulation.

## 4 Discussion

In the present study, we investigated the potential involvement of decreased Parkin expression following BMP4 stimulation in mitochondria-related abnormalities in diabetes-associated renal injury, using STZ-induced diabetic mouse kidneys and cultured mesangial cells as models. In both models, we observed a reduction in Parkin expression and downregulation of mitochondrial homeostasis-related genes such as *Ppargc1a* (PGC-1α), *Tfam*, *Cox4i1*, and *Atp5a1*. In STZ-induced diabetic mouse kidneys, the mtDNA copy number and COX activity were decreased, whereas BMP4-exposed mesangial cells additionally showed reduced ATP production and mitochondrial membrane potential. Furthermore, Parkin overexpression in mesangial cells significantly ameliorated some of the alterations associated with BMP4 stimulation. These results support the possibility that decreased Parkin expression is at least partially involved in the mitochondria-related abnormalities induced by BMP4 stimulation in mesangial cells.

Parkin plays a crucial role in mitochondrial quality control (11). Clinical and experimental studies have reported reduced Parkin expression or impaired PINK1/Parkin signaling in DN, which contributes to mitochondrial abnormalities and renal injury progression (12–14). Our findings obtained from mesangial cells are consistent with these previous reports and suggest that Parkin reduction may be one of the molecular alterations associated with the disruption of mitochondrial homeostasis in DN. Consistent with this, high glucose has been reported to reduce PGC-1α (26) and PINK1/Parkin (27) in mesangial cells. Moreover, mitochondrial dysfunction has been reported in extrarenal pathologies associated with CKD (23, 28); thus, impaired energy metabolism is considered important for understanding the pathophysiological significance of mitochondrial damage in DN.

One important observation in this study is the demonstration of an association between BMP4, a pro-fibrotic factor, and Parkin, which is involved in mitochondrial quality control. Through a series of studies, we have consistently reported that BMP4 induces mesangial matrix expansion (such as the production of type IV collagen) via the ALK-Smad1 pathway (4–6); however, its relationship with intracellular energy metabolism failure remains unclear. In the present study, BMP4 stimulation induced a decrease in Parkin expression and downregulation of mitochondrial homeostasis-related genes, and these alterations were partially ameliorated by BMP signaling inhibition and Parkin overexpression. These results indicate that BMP4 may be associated not only with its previously reported stimulatory effect on extracellular matrix production but also with mitochondria-related abnormalities accompanied by Parkin reduction. As such, BMP4 may be involved in both mesangial matrix expansion and disruption of mitochondrial homeostasis, indicating involvement in both structural and metabolic alterations. The inhibitor used, LDN-193189, selectively inhibits the BMP type I receptors (ALK2/ALK3) at the concentration used (19); accordingly, the suppression of BMP4-induced ECM gene expression by LDN-193189 (**Figure 4A**) confirms effective inhibition of BMP signaling. Notably, although BMP4 reduced endogenous Parkin at both the mRNA and protein levels (**Figures 1**, **2**), Parkin expressed from a constitutive promoter was not clearly reduced by BMP4 (**Figures 4E, F**), consistent with the decrease in *Park2* mRNA; because overexpression may alter the mode of regulation, this does not exclude post-transcriptional or protein-level mechanisms. This mRNA decrease was ameliorated by ALK inhibition (**Figure 4B**), implicating ALK signaling.

The cell-type distribution of BMP4 in the diabetic glomerulus has been characterized previously, including by our group. In previously reported wild-type STZ-induced diabetic models, BMP4 localizes predominantly to the mesangial region, colocalizing with type IV collagen but not with podocin (5), with only partial podocyte overlap (29); its type I receptor ALK3 is likewise mainly mesangial (4). Prominent podocyte localization has been reported mainly in genetically modified models (4, 29). These observations indicate that the mesangium is a principal site of BMP4 action, consistent with the mesangial focus of the present study and supporting its proposed role as an upstream factor in mesangial mitochondrial abnormalities.

From a therapeutic perspective, because Parkin acts as an intracellular E3 ubiquitin ligase, interest has focused on restoring its expression and Parkin-mediated mitophagy rather than on supplementing the protein itself. In DN, mitochondria-targeted interventions have attracted attention in this regard: the mitochondria-targeted antioxidant MitoQ restores high-glucose-reduced PINK1/Parkin expression via the Nrf2/PINK1 pathway and ameliorates renal injury (13), and FoxO1 activation promotes PINK1/Parkin-mediated mitophagy and protects diabetic podocytes (14). These findings raise the possibility that restoring mitochondrial homeostasis, with concomitant recovery of Parkin function, may attenuate the mitochondrial dysfunction associated with the BMP4-Parkin axis; whether such interventions also influence upstream BMP4 expression remains to be determined.

This study had several limitations. First, the association between Parkin reduction and mitochondrial homeostasis-related changes was assessed mainly at the level of mRNA expression and functional phenotypes; the intermediate molecular processes connecting them—including how Parkin indirectly regulates PGC-1α via ubiquitination of substrate proteins (10)—were not directly examined. Although the partial amelioration of BMP4-induced changes by Parkin overexpression supports the involvement of Parkin reduction, its contribution compared with other pathways cannot be determined from this study alone. Second, BMP signaling was evaluated solely by pharmacological inhibition, so the specific receptor subtypes and downstream molecules involved in Parkin reduction were not identified; although the inhibitor is selective for BMP type I receptors, the canonical TGF-β/Smad2/3 pathway was not directly assessed. Third, the temporal relationship between Parkin reduction and mitochondrial dysfunction, as well as the effects of *in vivo* interventions, were not examined; thus, clarifying its positioning within the progression of this pathology remains a challenge for future research. Furthermore, the *in vivo* analysis targeted the entire renal tissue and did not directly demonstrate the cell type specificity of the observed molecular changes, particularly those in mesangial cells. Additionally, although Parkin is known to mediate mitochondrial quality control, we did not directly evaluate mitophagy flux; therefore, our findings primarily reflect alterations in mitochondrial homeostasis-related indices. Fourth, as a short communication, this study provides an initial investigation into the relationship between BMP4 stimulation, Parkin reduction, and mitochondria-related abnormalities; the relative contribution of this pathway to the overall pathology of DN and its crosstalk with other pathological signals requires further investigation. Fifth, although mitochondrial dysfunction is intrinsically linked to oxidative stress and inflammation, we focused on mitochondrial homeostasis and bioenergetics; while cellular antioxidant capacity decreased, specific reactive oxygen species and inflammatory cytokines were not quantified, and their precise interplay with BMP4-induced Parkin downregulation remains for future study. In addition, although we confirmed that high glucose impairs mesangial mitochondrial function, a direct comparison between high-glucose and BMP4 stimulation, and an epistasis analysis combining high glucose with BMP4 inhibition, were not performed. Furthermore, whether ALK inhibition restores endogenous Parkin protein was not directly examined. Finally, identifying the downstream transcription factors mediating BMP4-induced Parkin downregulation was beyond the scope of this study and remains a subject for future work.

## 5 Conclusions

In conclusion, our findings suggest that decreased Parkin expression associated with BMP4 stimulation may be one of the novel molecular alterations contributing to mitochondria-related abnormalities in mesangial cells. The disruption of mitochondrial homeostasis accompanied by decreased Parkin expression may represent a candidate therapeutic pathway in DN. In this regard, interventions that restore mitochondrial homeostasis, including maintenance of Parkin and mitochondrial activation, may warrant further investigation as therapeutic strategies.

## Data Availability

The raw data supporting the conclusions of this article will be made available by the authors, without undue reservation.
