# Supplementary material for: Bone morphogenetic protein 4-associated mitochondrial dysfunction in diabetic nephropathy involves Parkin downregulation
Source: Front Clin Diabetes Healthc. 2026 Aug 11;7:1860424. doi: 10.3389/fcdhc.2026.1860424 (PMC13503189; doi:10.3389/fcdhc.2026.1860424)
Supplement: Supplementary file 1 [file Table1.docx]

Supplementary Material

**Supplementary Table 1.** Primer sequences used in this study

| **Gene** | **Forward Primer (5' → 3')** | **Reverse Primer (5' → 3')** | **Reference** |
| --- | --- | --- | --- |
| *Col1a1* | CCTCAGGGTATTGCTGGACAAC | CAGAAGGACCTTGTTTGCCAGG | Lin et al., 2017 |
| *Col4a1* | ATGGCTTGCCTGGAGAGATAGG | TGGTTGCCCTTTGAGTCCTGGA | Lin et al., 2017 |
| *Bmp4* | CGAGCCAACACTGTGAGGAGTT | GCTGCTGAGGTTGAAGAGGAAA | Matsubara et al., 2015. |
| *Pink1* | GCTTGCCAATCCCTTCTATG | CTCTCGCTGGAGCAGTGAC | Zhou et al., 2007 |
| *Park2* | TGGAAAGCTCCGAGTTCAGT | CCTTGTCTGAGGTTGGGTGT | Ma et al., 2023 |
| *Ppargc1a* | CCGTAAATCTGCGGGATGATG | CAGTTTCGTTCGACCTGCGTAA | Tamaki et al., 2015 |
| *Tfam* | TGAAGCTTGTAAATGAGGCTTGGA | CGGATCGTTTCACACTTCGAC | Tamaki et al., 2015 |
| *Nd1* | CTAGCAGAAACAAACCGGGC | CCGGCTGCGTATTCTACGTT | Quiros et al., 2018 |
| *Cox4i1* | ACTGCGCTCGTTCTGATTTGG | CATTCGCTTGGTCTGCATGG | Tamaki et al., 2015 |
| *Atp5a1* | ATGTGTCCGCTTACATTCCAACAA | GATCCGACACGGGACACAGA | Tamaki et al., 2015 |
| *Actb* | TGACAGGATGCAGAAGGAGA | GCTGGAAGGTGGACAGTGAG | Tamaki et al., 2015 |
| *Hk2* | GCCAGCCTCTCCTGATTTTAGTGT | GGGAACACAAAAGACCTCTTCTGG | Quiros et al., 2018 |

**Supplementary Figure S1.** High glucose impairs mitochondrial function in mesangial cells. (A and B) Cultured mesangial cells were exposed to normal glucose (NG), high glucose (HG, 30 mM), or NG plus mannitol (osmotic control) for 48 h. (A) ATP production and (B) mitochondrial membrane potential (ΔΨ_m_, JC-1). Data are presented as the mean ± SEM of three independent experiments. **P < 0.01 vs. NG; #P < 0.05 vs. HG.

**Supplementary Figure S2.** Representative JC-1 fluorescence images of cultured mesangial cells treated with vehicle (Control), BMP4 (15 ng/mL), or BMP4 + LDN-193189 (LDN) for 24 h. Red J-aggregates indicate polarized mitochondria, and green monomers indicate depolarized mitochondria. Images were acquired under blinded conditions using identical exposure and detector settings across all groups and displayed with identical linear settings (Keyence BZ-9000, PlanApo 20×/NA 0.75; merged in ImageJ v1.54). Scale bar, 20 µm.
